# Can biased search results change people’s opinions about anything at all? a close replication of the Search Engine Manipulation Effect (SEME)

**DOI:** 10.1371/journal.pone.0300727

**Published:** 2024-03-26

**Authors:** Robert Epstein, Ji Li

**Affiliations:** American Institute for Behavioral Research and Technology, Vista, California, United States of America; National Institute of Technology Silchar, India, INDIA

## Abstract

In previous experiments we have conducted on the Search Engine Manipulation Effect (SEME), we have focused on the ability of biased search results to shift voting preferences. In three new experiments with a total of 1,137 US residents (mean age = 33.2), we sought to determine whether biased search rankings could shift people’s opinions on topics that do not involve candidates or elections. Each of the new experiments looked at a different topic, and participants were pre-screened to make sure they didn’t have strong opinions about these topics. The topics were: Is artificial intelligence useful or dangerous? Is fracking helpful or dangerous? And: Are people born gay or do they choose to be gay? All participants were first asked various demographic questions, then shown brief summaries of the “pro” and “anti” views on each topic, and then asked their opinions about each topic. Next, participants were allowed to conduct an online search using our mock search engine (Kadoodle) lasting up to 15 minutes. In each experiment, one-third of the participants saw biased search results favoring one perspective; one-third saw biased search results favoring the opposing perspective; and one-third (the control group) saw mixed search results. After completing their search, participants were again asked for their opinions about the topic. Our primary dependent variable was Manipulation Power (MP), the percentage increase in the number of participants favoring one viewpoint after having viewed search rankings favoring that viewpoint. The MPs in the three experiments were 25.0%, 30.9%, and 17.8%, respectively. Corresponding shifts were also found for how persuasive participants found each viewpoint to be and for how much they trusted each viewpoint. We conclude that search rankings favoring one viewpoint on a wide range of topics might be able to cause people who have not yet formulated a strong opinion on such topics to adopt the favored perspective. If our findings prove to be robust, we are exposing what might be considered an unforeseen consequence of the creation of search engines, namely that even without human interference, search algorithms will inevitably alter the thinking and behavior of billions of people worldwide on perhaps any topic for which they have not yet formed strong opinions.

## 1. Introduction

Controlled experiments conducted in recent years have shown that bias in search engine results can rapidly shift the opinions and voting preferences of undecided voters–by as much as 80% in some demographic groups [1]. Research has also shown that this effect–the search engine manipulation effect (or SEME, pronounced “seem”)–can easily be masked so that no users are aware of the bias they are seeing [1]. SEME has been partially or fully replicated multiple times since it was first published in 2015 [2–16].

Research has also shown that when people do suspect that they are viewing biased search results, that awareness does not necessarily protect them from the impact of the bias. Epstein and Robertson [1] showed in a controlled experiment with more than 2,000 participants from all 50 US states that the few people (8.6%) who could recognize the bias shifted even farther, on average, than people who did not recognize the bias. Why this occurred is not clear, but it could have been because people have inordinate faith in the validity of computer output, at least in part because they have no idea how computers work [17,18]. Research has also shown that vulnerability to SEME and other new forms of online manipulation varies substantially from one demographic group to another [1,6–12].

At least three other features of search engines make them potentially problematic, at least in the eyes of some experts and public policy makers: First, all the content shown to users on search engines is ephemeral. Search suggestions, answer boxes, and search results are all generated on the fly, impact users, and then disappear, and online ephemeral content can impact people’s thinking significantly [19,20]. Because they are not stored anywhere, they leave no paper trail for authorities to trace. If biased search results shifted votes in an election–perhaps, in a close election, so many votes that that bias determined the outcome–there would be no way to go back in time to document such an effect [21,22,cf. 23].

Second, over the years, search engines have based their content on increasingly vast amounts of data they have collected about each user; in other words, they now personalize (or “customize”) content to meet the needs of individual users [24]. Many users like this feature of modern search engines, which they consider to be the digital equivalent of personal shoppers [25,26]. On the downside, a long history of research in the marketing and advertising fields has shown that the more one knows about the customer, the easier it is to manipulate him or her [27,28]. This ability applies as much to voters as it does to shoppers [29,30].

Third, because about 92% of search worldwide–everywhere outside of mainland China (the PROC)–is conducted on just one search engine, with no other search engine attracting more than 4% of search [31,32,cf. 33], if the leading search engine chooses to shift votes or opinions in just one direction, there is no way to counteract that very powerful form of influence. If the bias has been masked, there also may be no way to detect it. This is very different from most forms of influence that affect people every day, especially in the days leading up to elections. Most forms of influence–billboards, television and radio commercials, newspapers ads and editorials, online ads and podcasts–are inherently competitive. If you have the resources, you counter your opponent’s brutal attack ad with one or more ads that are even more brutal. But if the dominant search engine chooses–either by deliberate acts of its employees or by unconscious or neglectful management of its algorithms [20,22,34]–to support one political candidate or party, there is no way to counteract that influence.

For these reasons, it is important to understand how SEME works, who it affects, and the magnitude of its ability to alter opinions, beliefs, purchases, behavior, and votes. As we have been arguing elsewhere in recent years, it is also important that we develop permanent monitoring systems that can preserve and analyze ephemeral content on a large scale [35–37]. If we don’t preserve ephemeral content, we will never know how and to what extent existing and emerging tech companies are impacting our minds, our children, and our political systems.

Nearly all the research that has been conducted on SEME has focused on only one of these domains–namely, the ability of biased search results to alter the opinions and voting preferences of undecided voters. We are aware of one conference presentation in which SEME was partially replicated in a context involving people’s knowledge about health issues [38]. An earlier study found that search results linked to webpages that contained high-quality information about vaccines communicated more knowledge to people than did search results linked to low-quality webpages [39], but that study did not measure opinion shifts.

We are left with a consequential question that we believe has not yet been answered adequately: Can biased search results shift people’s opinions not just about political candidates but about a wide range of different topics–perhaps any topic at all? We acknowledge that this question applies mainly, if not exclusively, to people who have not yet made up their mind about that topic: about where to go on vacation, about what kind of car they should buy, about whether gays should be able to marry or adopt children, and so on. How much power do biased search results have, across a wide range of different topics and issues, to shift the opinions and behavior of people who are vulnerable to being influenced?

## 2. Experiment 1: Can biased search results shift people’s views about artificial intelligence (AI)?

### 2.1 Methods

#### 2.1.1 Ethics statement

The federally registered Institutional Review Board (IRB) of the sponsoring institution (American Institute for Behavioral Research and Technology) approved this study with exempt status under HHS rules because (a) the anonymity of participants was preserved and (b) the risk to participants was minimal. The IRB is registered with OHRP under number IRB00009303, and the Federalwide Assurance number for the IRB is FWA00021545. Informed written consent was obtained for all three experiments as specified in the Procedure section below.

#### 2.1.2 Participants

378 participants were recruited online from the Amazon Mechanical Turk (MTurk) subject pool during March 2016. The mean age of our participants was 33.8 (*SD* = 11.4). 56.3% (n = 213) of our participants identified themselves as female and 43.7% (n = 165) as male. For detailed information about the basic demographic characteristics of our participants in all three experiments, see S1 Table.

91.3% (n = 345) of our participants reported using Google as their primary search engine; 4.2% (n = 16) reported using Bing, 2.6% (n = 10) reported using Yahoo, and 1.9% (n = 7) reported using some “other” search engine. Participants reported conducting a wide range of number of searches per week–from 1 per week to over 200 per week (*M* = 12.8, *SD* = 17.1). 44.2% (n = 167) of participants identified themselves as politically liberal, 30.2% (n = 114) as moderate, 17.7% (n = 67) as conservative, 6.6% (n = 25) as having no political viewpoint, and 1.3% (n = 5) as other.

We asked participants how familiar they were with arguments that favored the use of artificial intelligence (AI) and arguments that were critical of AI on a scale from 1 to 10, where 1 represented “Not familiar at all” and 10 represented “Very familiar.” The mean familiarity level with pro-AI arguments was 3.1 (*SD* = 2.2), and the mean familiarity level with anti-AI arguments was 3.1 (*SD* = 2.3).

#### 2.1.3 Procedure

The experiment was conducted online and employed a pre/post design. Participants were first asked, “Do you have strong beliefs about artificial intelligence?” and only people who clicked “No” were allowed to continue. Then participants were given basic instructions and asked for their informed consent (S5 Text). As required by the sponsoring institution’s IRB, participants were not asked for identifying information such as name, email address, or telephone number. The participants were then asked a series of demographic questions. They were shown brief (about 100 words) paragraphs about AI. The first paragraph presented a point of view favoring AI, and the second presented a point of view opposing AI (see S2 Text for the full content).

Participants were then asked six opinion questions about AI: two regarding their overall impressions, two regarding how persuasive they found the two viewpoints they had read, and two regarding how much they trusted those two viewpoints; for the full text of the questions, which participants answered on 10-point Likert scales, see S1 Fig. Then participants were asked two choice questions: First, on an 11-point scale from 5 to 0 to 5 (S1 Fig), participants indicated which viewpoint they favored, with “Pro AI” and “Anti AI” appearing at each end of the scale, with the positions counterbalanced. Finally, participants were asked to choose which viewpoint they favored in a forced-choice question (S1 Fig)–again, with the positions of the answers counterbalanced. This page of questions comprised the pre-search test.

**Fig 1:**
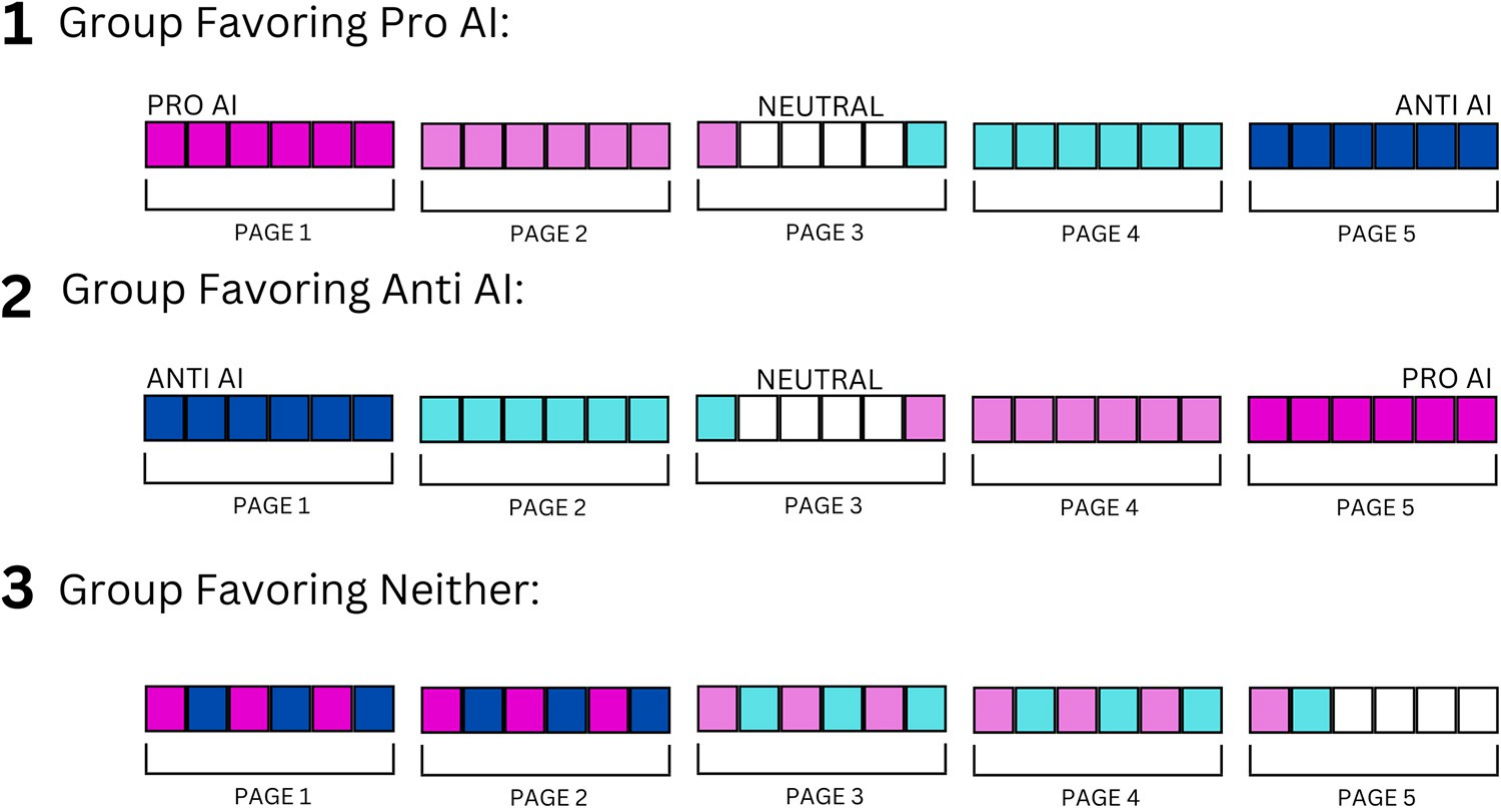
Ordering of search results for the three groups. Each small square represents a search result, and each group of six squares represents the search results on one page. Dark pink signifies that a search result links to a web page in which the content has been rated (by independent raters) to be pro-AI. Light pink signifies that search results link to web pages that are less favorable to AI. White signifies that the linked web pages are relatively neutral toward AI. Light blue signifies that search results link to web pages that are somewhat anti-AI. Medium blue signifies that search results link to web pages that are strongly anti-AI. In Group 1 (Pro AI), the search results are in order from pro-AI to anti-AI. In Group 2 (Anti-AI), the search results are in the opposite order. In Group 3 (Control), pro- and anti-AI search results alternate.

At the beginning of the experiment, all participants were randomly assigned to one of three groups: Pro-AI, Anti-AI, or the Control Group, in which people saw alternating pro- and anti-AI arguments. The sequences are shown in Fig 1.

All 30 webpages used in this experiment had previously been rated by five independent reviewers on an 11-point scale from 5 to 0 to 5, where “Pro AI” and “Anti AI” appeared at either end of the scale, and their order was counterbalanced. Based on the mean ratings of the reviewers, the search results were ranked from the most Pro AI (referring to the web page to which the search result linked) to the most Anti AI (again, referring to the web page to which the search result linked), with the relatively neutral search results in the middle (Fig 1, Group 1).

**Fig 2:**
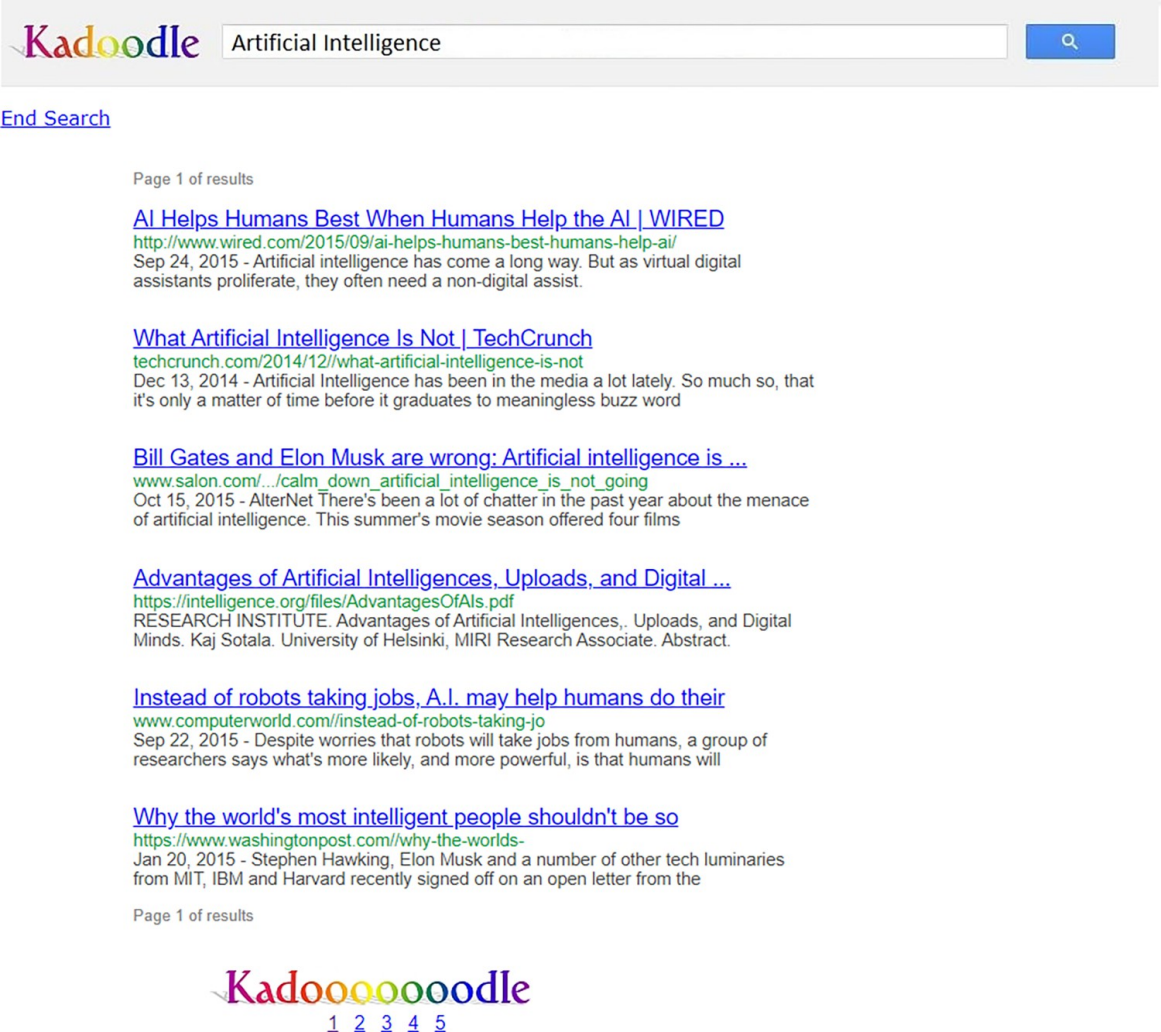
Example of Kadoodle search results page. Each search results webpage contained six different search results. The participant could click on a link to view the corresponding web page, or he or she could click on one of the numbers at the bottom of the page to switch to a different page of search results. The “End Search” shortcut can be seen in the top left corner of the page.

After participants answered those eight questions (six opinion questions and two choice questions) about the pro- and anti-AI points of view, they were then given up to 15 min to use our Kadoodle search engine–a Google simulator–to learn more about AI. Our search engine showed participants five pages of search results, with six search results per page. Participants could click on any of the results and could switch between the pages by clicking on numbers at the bottom of each page (see Fig 2).

All search results were real, scraped from the Google search engine, and all webpages were real, scraped from the internet. The webpages were presented as image files created from the original pages, with no active links.

Participants could end their search by clicking a button on the top left of the screen that read “End Search” (Fig 2). If they failed to click the button, the search session would end when a 15-min time limit was reached.

Now participants were again asked to answer the six opinion questions and two choice questions they had answered prior to search (the post-search test). After participants responded to these questions, they were asked whether anything in the experiment had bothered them. If they answered “yes,” they could then explain what had bothered them in a text box. The purpose of asking participants about what bothered them was to determine whether they detected bias in the search results. We could not ask directly about bias because leading questions of that sort are known to inflate estimates [40].

Finally, participants were thanked for their participation in the experiment and provided with a code number that they could use to be paid by MTurk.

### 2.2 Results

Most of the experiments we have conducted on online manipulation since we began this type of research in 2013 [1,6–13] have used “vote manipulation power” (VMP) as the most informative metric of change. VMP was defined as the post-manipulation percentage increase in the number of people voting for the candidate favored in the manipulation (see S1 Text for further details). Because we are now extending our investigation to look at topics that are not election-related, we are introducing a broader variant of VMP, calling it simply “manipulation power” or MP. We define MP as the post-manipulation percentage increase in the number of people choosing the opinion favored in the manipulation (or the belief, candidate, product, perspective, or other categorical content that can be made to look superior to an alternative).

In Experiment 1, the MP was 25.0% (McNemar’s Test *X*^*2*^ = 22.22, *p* < 0.001), which means that in the two bias groups combined, the bias in the search results increased the number of people choosing either a pro-AI or an anti-AI perspective by 25.0%. Specifically, before the search was conducted, the total number of people in the two bias groups who chose the favored perspective was 124. After the search, that number increased by 25.0% to 155.

On the 11-point voting preference scale, pre-manipulation, we found no significant difference between the mean ratings in the three groups (*M*_Pro_ = 1.62, *SD* = 2.5; *M*_Anti_ = 1.78, *SD* = 2.4; *M*_Control_ = 1.31, *SD* = 2.5; Kruskal-Wallis *H* = 3.13; *p* = 0.209 NS). Post manipulation, we found a significant difference between mean ratings in the three groups (*M*_Pro_ = 1.99, *SD* = 2.7; *M*_Anti_ = 0.18, *SD* = 3.0; *M*_Control_ = 1.03, *SD* = 2.9; *H* = 24.68; *p* < 0.001). Participants in Group 1 shifted 0.37 points toward the favored opinion (Pro AI), and participants in Group 2 condition shifted 1.6 points towards the favored opinion (Anti AI). In addition, the pre-manipulation mean preference for the favored opinion (Groups 1 and 2 combined) was significantly different from the post-manipulation mean preference for the favored opinion (Groups 1 and 2 combined) (*M*_*Pre*_ = 0.004; *SD*_*Pre*_ = 3.0; *M*_*Post*_ = 0.96; *SD*_*Post*_ = 3.1; *M*_*Diff*_ = 0.956; Wilcoxon Signed Ranks *z* = 6.00; *p* < 0.001).

The shift was also indicated by three measures for each of the two opposing opinions: measures of overall impression, persuasiveness, and level of trust (S1 Fig). Pre to post, the mean favored opinions increased for all three measures, and the non-favored opinions decreased for all three measures. Pre to post, the overall change in opinions was highly significant for all three measures and was in the predicted direction (Table 1).

**Table 1:** Experiment 1: Pre- and post-search opinion ratings of the favored and non-favored candidate measured on 10-point scales, bias groups only.

	Favored Opinion Mean (*SD*)			Non-Favored Opinion Mean (*SD*)				
	Pre	Post	Diff	Pre	Post	Diff	*z* ^†^	*p*
**Overall Impression**	5.65 (2.6)	6.46 (2.5)	+ 0.81	5.74 (2.6)	4.98 (2.4)	- 0.76	-5.20	< 0.001
**Persuasiveness**	5.63 (2.4)	6.88 (2.5)	+ 1.25	5.76 (2.3)	5.09 (2.6)	- 0.67	-6.46	< 0.001
**Level of Trust**	5.61 (2.2)	6.63 (2.3)	+ 1.02	5.85 (2.1)	5.36 (2.4)	- 0.49	-6.15	< 0.001

^†^The z values come from Wilcoxon Signed Ranks Test between the post-search minus pre-search ratings for the favored candidate and the post-search minus pre-search ratings for the non-favored candidate.

In the two bias groups combined (Groups 1 and 2, *n* = 246), the number of people who noticed bias in the search results they saw was 38.2%. This is consistent with the level of bias perception in other SEME experiments when masking has not been employed to disguise the bias [1,4–6].

## 3. Experiment 2. Can biased search results shift people’s views about fracking?

### 3.1 Methods

#### 3.1.1 Participants

394 participants were recruited online from MTurk during March 2016. The mean age of our participants was 32.9 (*SD* = 10.2). 52.0% (n = 205) of our participants identified themselves as female and 48.0% (n = 189) as male. For detailed information about basic demographic characteristics, see S1 Table.

94.2% (n = 371) of the participants selected Google as their primary search engine, 4.1% (n = 16) as Bing, 1.5% (n = 6) as Yahoo, and 0.3% (n = 1) as other. Participants reported the number of searches they conducted per week ranging from 0 to 150 (*M* = 14.4, *SD* = 18.1). 42.4% (n = 167) of participants reported being liberal, 33.8% (n = 133) as moderate, and 15.5% (n = 61) as conservative; 6.1% (n = 24) reported having no political views, and 2.3% (n = 9) reported their political viewpoint as other. The mean familiarity level of participants with pro-fracking arguments was 3.6 (*SD* = 2.3); for anti-fracking arguments, it was 4.2 (*SD* = 2.5).

#### 3.1.2 Procedure

The procedure for Experiment 2 was the same as for Experiment 1, except that the topic was fracking.

### 3.2 Results

In Experiment 2, the MP was 30.9% (McNemar’s Test *X*^*2*^ = 25.14, *p* < 0.001), which means that in the two bias groups combined, the bias in the search results increased the number of people choosing either a pro-fracking or an anti-fracking perspective by 30.9%. Specifically, before the search was conducted, the total number of people in the two bias groups who chose the favored perspective was 136. After the search, that number increased by 30.9% to 178.

On the 11-point voting preference scale, pre-manipulation, we found no significant difference between the mean ratings in the three groups (*M*_Pro_ = -0.79, *SD* = 2.7; *M*_Anti_ = -0.68, *SD* = 2.7; *M*_Control_ = -0.24, *SD* = 2.7; *H* = 2.48; *p* = 0.289 NS). Post manipulation, we found a significant difference between mean ratings in the three groups (*M*_Pro_ = -0.09, *SD* = 3.2; *M*_Anti_ = -2.44, *SD* = 2.8; *M*_Control_ = -0.97, *SD* = 3.7; *H* = 40.35; *p* < 0.001). Participants in Group 1 shifted 0.7 points toward the favored opinion (Pro Fracking), and participants in Group 2 condition shifted 1.76 points towards the favored opinion (Anti Fracking). In addition, the pre-manipulation mean preference for the favored opinion (Groups 1 and 2 combined) was significantly different from the post-manipulation mean preference for the favored opinion (Groups 1 and 2 combined) (*M*_*Pre*_ = -0.09; *SD*_*Pre*_ = 2.8; *M*_*Post*_ = 1.12; *SD*_*Post*_ = 3.3; *M*_*Diff*_ = 1.21; *z* = 8.06; *p* < 0.001).

The shift was also indicated by three measures for each of the two opposing opinions: measures of overall impression, persuasiveness, and level of trust (S1 Fig). Pre to post, the mean favored opinions increased for all three measures, and the non-favored opinions decreased for all three measures. Pre to post, the overall change in opinions was highly significant for all three measures and was in the predicted direction (Table 2).

**Table 2:** Experiment 2: Pre- and post-search opinion ratings of the favored and non-favored candidate measured on an 11-point scale, bias groups only.

	Favored Opinion Mean (*SD*)			Non-Favored Opinion Mean (*SD*)				
	Pre	Post	Diff	Pre	Post	Diff	*z* ^†^	*p*
**Overall Impression**	5.73 (2.3)	6.68 (2.6)	+ 0.95	5.78 (2.3)	4.80 (2.6)	- 0.98	-7.66	< 0.001
**Persuasiveness**	5.77 (2.2)	6.95 (2.5)	+ 1.18	5.66 (2.3)	4.75 (2.7)	- 0.91	-7.47	< 0.001
**Level of Trust**	5.44 (2.0)	6.26 (2.6)	+ 0.82	5.49 (2.1)	4.77 (2.6)	- 0.72	-6.56	< 0.001

^†^ The z values come from Wilcoxon signed ranks test between post-search minus pre-search ratings for the favored candidate and the post-search minus pre-search ratings for the non-favored opinion.

In the two bias groups combined (Groups 1 and 2, *n* = 286), the number of people who noticed bias in the search results they saw was 50.3%. This is higher than the typical level of bias perception we have found in other SEME experiments when masking has not been employed to disguise the bias [1,4–6].

## 4. Experiment 3. Can biased search results shift people’s views about sexual orientation?

### 4.1 Methods

#### 4.1.1 Participants

365 participants were recruited online from MTurk during March 2016. The mean age of our participants was 32.8 (*SD* = 10.6). 55.9% (n = 204) of our participants identified themselves as female and 44.1% (n = 161) as male. For detailed information about basic demographic characteristics, see S1 Table.

93.7% (n = 342) of our participants reported Google as their primary search engine, 2.5% (n = 9) as Bing, 2.2% (n = 8) as Yahoo, and 1.6% (n = 6) as other. Participants reported the number of searches they conducted per week ranging from 0 to 250 (*M* = 14.1, *SD* = 24.6). 42.7% (n = 156) of participants reported being liberal, 36.7% (n = 134) as moderate, and 12.1% (n = 44) as conservative; 5.8% (n = 21) reported having no political views, and 2.7% (n = 10) reported their political viewpoint as other. The mean familiarity level of participants with born-gay arguments was 7.3 (*SD* = 2.6); for choose-to-be-gay arguments, it was 7.3 (*SD* = 2.5).

#### 4.1.2 Procedure

The procedure for Experiment 3 was the same as for Experiment 1, except that the topic was sexual orientation–specifically, whether people are born gay or whether they choose to be gay.

### 4.2 Results

In Experiment 3, the MP was 17.8% (McNemar’s Test *X*^*2*^ = 11.81, *p* < 0.001), which means that in the two bias groups combined, the bias in the search results increased the number of people choosing either a pro-AI or an anti-AI perspective by 17.8%. Specifically, before the search was conducted, the total number of people in the two bias groups who chose the favored perspective was 135. After the search, that number increased by 17.8% to 159.

On the 11-point voting preference scale, pre-manipulation, we found no significant difference between the mean ratings in the three groups (*M*_Born_ = 1.54, *SD* = 2.9; *M*_Choose_ = 1.27, *SD* = 3.1; *M*_Control_ = 1.86, *SD* = 2.9; *H* = 2.25; *p* = 0.325 NS). Post manipulation, we found a significant difference between mean ratings in the three groups (*M*_Born_ = 2.56, *SD* = 3.0; *M*_Choose_ = 0.41, *SD* = 3.6; *M*_Control_ = 2.01, *SD* = 3.2; *H* = 26.87; *p* < 0.001). Participants in Group 1 shifted 1.02 points toward the favored opinion (Born Gay), and participants in Group 2 condition shifted 0.86 points towards the favored opinion (Choose to be Gay). In addition, the pre-manipulation mean preference for the favored opinion (Groups 1 and 2 combined) was significantly different from the post-manipulation mean preference for the favored opinion (Groups 1 and 2 combined) (*M*_*Pre*_ = 0.12; *SD*_*Pre*_ = 3.3; *M*_*Post*_ = 1.05; *SD*_*Post*_ = 3.6; *M*_*Diff*_ = 0.93; *z* = 7.13; *p* < 0.001).

The shift was also indicated by three measures for each of the two opposing opinions: measures of overall impression, persuasiveness, and level of trust (S1 Fig). Pre to post, the mean favored opinions increased for all three measures, and the non-favored opinions decreased for all three measures. Pre to post, the overall change in opinions was highly significant for all three measures and was in the predicted direction (Table 3).

**Table 3:** Experiment 3: Pre- and post-search opinion ratings of the favored and non-favored candidate measured on an 11-point scale, bias groups only.

	Favored Opinion Mean (*SD*)			Non-Favored Opinion Mean (*SD*)				
	Pre	Post	Diff	Pre	Post	Diff	*z* ^†^	*p*
**Overall Impression**	6.00 (2.6)	6.65 (2.9)	+ 0.65	5.93 (2.7)	5.24 (3.0)	- 0.69	-5.97	< 0.001
	**Persuasiveness**	5.70 (2.8)	6.63 (3.1)	+ 0.93	5.69 (2.8)	4.87 (3.1)	- 0.82	-7.91	< 0.001
	**Level of Trust**	5.77 (2.7)	6.52 (3.0)	+ 0.75	5.82 (2.8)	5.08 (3.1)	- 0.74	-6.90	< 0.001

^†^The z values come from Wilcoxon Signed Ranks Test between the post-search minus pre-search ratings for the favored candidate and the post-search minus pre-search ratings for the non-favored candidate.

In the two bias groups combined (Groups 1 and 2, *n* = 252), the percentage of people who noticed bias in the search results they saw was 28.6%. This is similar to the typical level of bias perception we have found in other SEME experiments when masking has not been employed to disguise the bias [1,4–6].

## 5. Discussion

In the three experiments we have described above, we produced shifts in preferences of 25.0%, 30.9%, and 17.8%, respectively, after our participants conducted just one search on our Kadoodle search engine. These numbers are based on shifts in the two bias groups combined. The fact that participants were randomly assigned to one or the other of those two groups means we were able to shift people’s thinking for or against a particular perspective arbitrarily. Mean voting preferences on an 11-point scale and mean opinion ratings also shifted predictably in the direction of the bias. These results support our conjecture that bias in the online search results displayed to users by search engine companies have the potential to change people’s thinking about–well, perhaps anything at all.

Key to this finding is the fact that we deliberately worked with people who did not, to begin with, already have strong opinions about the three topics we explored. Presumably, people with strong opinions about such matters would be difficult to influence with biased search results [e.g., 41]. That is a matter we are continuing to investigate in ongoing research. If indeed people who are undecided on some issue are the most vulnerable to such a manipulation, it is notable that search engine companies are not only in a unique position to employ bias in search results to impact people’s thinking, they are also in a unique position to identify people who are most vulnerable to this type of manipulation–that is, people who have not yet made up their minds. A company such as Google, which openly tracks people through their emails [42] (using Gmail, the most widely used email system in the world [43]), online searches (using the Google search engine, which handles 92% of online search in most countries [31,32]), Chrome (the most widely used browser in the world [44]), Android (the most widely used mobile operating system in the world [45]), and many other platforms and applications [46,47], can easily identify people who are undecided or uncommitted on some issue.

### 5.1 Conclusions

Even without intent by employees or executives at Google and other companies that operate search engines, the power that biased search results appear to have to shift opinions about a wide range of topics should be a matter of great concern to legislators, regulators, and public policy makers. We make this strong assertion because, by definition, search algorithms always do three things: they *filter* content (by selecting a small amount of content to display while setting aside a vast amount of other content), they *order* content (by ranking the content they will display), and they *customize* content (by adjusting both the filtering and ordering to best match the interests and needs of the user). In other words, in some sense *all* search results are biased, and therein lies their value. For a given user, search results will always favor one dog food over another, and we wouldn’t want it any other way. The problem is that for people sitting on a fence, that customized, filtered, and ranked content the search engine shows them appears to be effective as a tool for pushing people to tumble off one side of that fence.

To put this another way, the search engine is not only the most powerful tool ever invented for providing factual answers to simple questions, it might also be the most powerful tool ever invented for influencing people’s opinions, even if influence was never the intent. In a separate study [48] we examined this issue from the perspective of operant conditioning. About 86% of the searches people conduct on major search engines are for simple facts [49], and those facts almost invariably turn up in the top position of search results. Like rats in a Skinner box, we thus learn, over and over again, that the most valuable and accurate search results are the ones at the top of the list. When that day comes when we pose an open-ended query–“best restaurant in Atlanta,” “is fracking safe?,” or “how to solve the immigration problem”–we again tend to attend to and trust those high-ranking links, which will bring us to web pages that likely favor one perspective. That should surprise no one; there are no equal-time rules in search algorithms, after all. They are designed to find the “best” results, not to show a series of pro- and anti- results in alternating order (like the results we showed in our Control Group). Google does so by examining link patterns [50], but no matter what technique is used, a search algorithm will always, or nearly always, tend to favor one perspective over another. That favoritism might occur because one perspective is dominant on the internet, because of the conscious or unconscious biases of the programmers who created and maintain the search algorithm [51–53], or because of company policies that elevate or suppress content deliberately through white listing or black listing [34,54]. The present study extends previous research only in helping to shed light on one issue: Could search engine bias shift people’s views about a wide range of different topics? The answer appears to be yes.

To put this issue yet another way: SEME is a list effect with a difference. Unlike other list effects researchers have studied over the past century, beginning with the serial position effect [55–57], SEME is supported by a daily regimen of operant conditioning that will never stop. Simple factual searches will continue to teach people *ad nauseum* that high-ranking search results are truer and more valid than lower ranking search results. Presumably this is why companies worldwide spend vast sums each year trying to push their products a notch or two higher in Google search results; a single increment can increase clicks by 32.3% [58].

In research we are currently conducting on what we call the “digital personalization effect” (DPE), we are learning that personalization–for example, showing people content from sources we know they trust–can dramatically increase the impact of SEME and other new forms of influence the internet has made possible [59]. When you combine three causal factors–(1) bias in search results, which is an essential and important feature of good search results, (2) customization in search results, which Google in particular has long taken pride in providing [24,60], and (3) a company’s ability to identify just those users who are especially vulnerable to influence–a troubling picture emerges. The picture becomes even more alarming when one recognizes that both search suggestions [11] and answer boxes [10]–both of which are commonly shown by Google search–also have the power to shift opinions. What if all of these factors align to push opinions in the same direction? And what if these types of influence are similarly biased in online experiences people are having day after day on multiple platforms? We are currently exploring these questions in experiments on what we call the “multiple exposure effect” (MEE) and the “multiple platforms effect” (MPE).

### 5.2 Limitations and future research

Our conclusions are subject to a number of constraints, two of which we believe are obvious and nontrivial. First, our subjects were drawn from the MTurk subject pool. In recent years, that subject pool has been tainted by bots [61,62], and concerns have been raised about just how representative the US portion of that subject pool is of the general population [63,64]. Fortunately, we conducted the present experiments in early 2016, well before most of the substantive concerns about MTurk were expressed [61,62]. Nevertheless, we acknowledge that the subjects in our experiments are not necessarily representative of the general population, a matter that can only be explored with replications using other sampling methods. On the bright side, our participants were demographically diverse (S1 Table)–far more so than the small group of sophomores at a single college or university who have so often been utilized in social science studies [65–67].

Second, we made no attempt to measure the long-term effects of the opinion shifts we measured. The impact we had on participants in our six bias groups might have been as ephemeral as search results typically are. Although our procedures can shed no light on this issue, we would be remiss in not pointing out that a search engine company such as Google could easily expose users to similarly biased content dozens or even hundreds of times over a period of a few months. If such exposures are additive in their impact, as our most recent research suggests [68], it is not unreasonable to believe that our experiments might be underestimating the power that biased search results might have on people’s thinking about virtually any topic (as long as the users have not already formed strong opinions).

Our Kadoodle simulator also differed from Google’s search engine in some respects. Google typically shows many pages of search results with about 10 results per page (on desktop and laptop computers). We showed only five pages of search results with only six results per page. We also did not show people search suggestions or answer boxes, which have become common on Google search pages in recent years. When answer boxes are added to search results, people spend less time examining search results and click on fewer search results [10]; if the answer boxes share the bias of the search results, however, opinions and votes shift even farther in the direction of the bias than they would have had only search results been shown [10]. Again, if search suggestions share the same bias as the search results, they too will increase the impact of those results [11]. So although our simulator differs from Google’s home page, it does so mainly in ways that make it less powerful as a source of influence.

Our “pro” and “anti” design could also be viewed as simplistic, although our 11-point scale–the first of our two choice questions–does allow participants to indicate intermediate degrees of support. We also acknowledge that we only evaluated three topics; this necessarily limits the generalizability of our results. The fact that we found different levels of opinion shifts for each of these three topics, ranging from 17.8% to 30.9%, suggests that bias in search results might, in general, have more power to influence people’s views about some topics than other topics. There are also undoubtedly individual differences in people’s vulnerability to such influence. Finally, we note that our data were collected in 2016. Given that both the form and function of search engines has changed little since then, we have no reason to believe that our findings would be difficult to replicate today. That said, it is important that our findings be replicated with contemporary groups.

Regarding future research, we have already mentioned three projects we have in progress that will shed more light on new forms of manipulation that the internet has made possible: MEE, MPE, and DPE. Regarding the range of opinions that might be influenced by biased (or by biased and personalized) search results, determining and understanding that range can be accomplished by varying topics in systematic ways. We have already learned that different demographic groups vary in how vulnerable they are to the manipulation we employed in the present experiments (S2 to S5 Tables), and we also have found demographic effects in other studies of online influence [1,9–11,13,59]. Further research might show predictable patterns in how vulnerable different demographic groups are (and, for that matter, in how vulnerable different individuals are) to having their opinions altered on different topics by biased search results. An extensive literature on influence and decision-making has already shown how demographic characteristics interact both with types of influence and the topics being considered [1,11,69–71].

Future research should also explore an odd feature of the search engine–one that we alluded to earlier and that might be considered a disturbing unforeseen consequence [72] of the creation of search engines–a consequence that is unavoidable, given the way search engines must work. Search results are useful precisely because they order information from best to worst; an equal-time rule would make them worthless, although perhaps–as a way of protecting the free-and-fair election from undue influence–an exception could be made someday for links to information about political candidates. Generally speaking, however, search results will *always* train people to value high-ranking results over lower ones, which means perforce that search results shown in response to open-ended queries will *always* shift people’s thinking and behavior, sometimes in trivial ways and sometimes in profound ones. This will almost always occur, moreover, without people’s awareness [1]. If shifts of this sort are deliberately programmed by software engineers, humanity might always be unduly influenced by such people. Our guess, though, is that only an infinitesimally small portion of open-ended queries are of interest to programmers or executives at tech companies. That means that the vast majority of shifts in opinions and behavior being produced by search engines 24 hours a day in people around the world are currently being determined by algorithms without human guidance.

Where algorithms are being left to their own devices (so to speak) by their human creators, they are currently determining what content goes viral or gets suppressed, what many people buy, what many people believe, and whom many people vote for. As generative AI systems are increasingly incorporated into the algorithms that currently dominate our lives, will the growing power of these systems be used in humanity’s interest? Will we even understand what is happening to us?

## Supporting information

S1 Fig Pre-search impression questions. (DOCX)

S1 Table Experiment 1, 2, & 3 demographics. (DOCX)

S2 Table Demographics analysis by gender. (DOCX)

S3 Table Demographics analysis by age. (DOCX)

S4 Table Demographics analysis by education level. (DOCX)

S5 Table Demographics analysis by ethnicity. (DOCX)

S1 Text Manipulation Power (MP) calculation. (DOCX)

S2 Text Artificial intelligence summary. (DOCX)

S3 Text Fracking summary. (DOCX)

S4 Text Sexual orientation summary. (DOCX)

S5 Text Request for informed consent. (DOCX)
