# Supplementary figures and images for: Can biased search results change people’s opinions about anything at all? a close replication of the Search Engine Manipulation Effect (SEME)
Source: PLoS One. 2024 Mar 26;19(3):e0300727. doi: 10.1371/journal.pone.0300727 (PMC10965084; doi:10.1371/journal.pone.0300727)

**S1 Fig: Pre-Search Impression Questions**

**
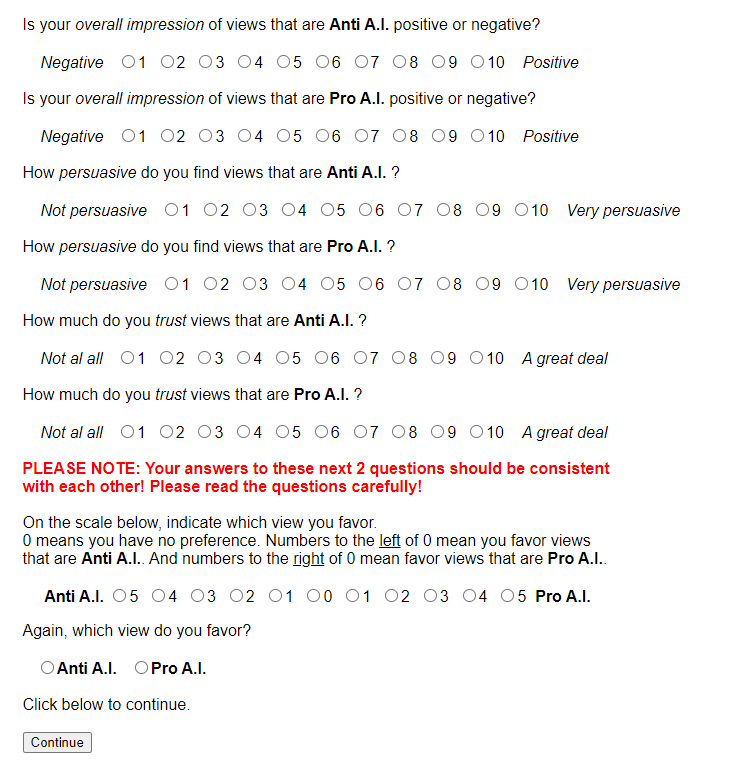
**

Supplement: S1 Fig — (DOCX) [file pone.0300727.s001.docx]
