# Supplementary material for: Can biased search results change people’s opinions about anything at all? a close replication of the Search Engine Manipulation Effect (SEME)
Source: PLoS One. 2024 Mar 26;19(3):e0300727. doi: 10.1371/journal.pone.0300727 (PMC10965084; doi:10.1371/journal.pone.0300727)
Supplement: S1 Table — (DOCX) [file pone.0300727.s002.docx]

**S1 Table: Experiment 1, 2, & 3 Demographics**

|  | **Experiment 1**  **(n = 378)** | **Experiment 2**  **(*n* = 394)** | **Experiment 3**  **(*n* = 365)** |
| --- | --- | --- | --- |
| **Mean Age (*SD*)** | 33.8 (11.4) | 32.9 (10.2) | 32.8 (10.6) |
| **Gender (%)** |  |  |  |
| Female | 213 (56.3%) | 205 (52.0%) | 204 (55.9%) |
| Male | 165 (43.7%) | 189 (48.0%) | 161 (44.1%) |
| **Education (%)** |  |  |  |
| None | 0 (0%) | 1 (0.3%) | 0 (0%) |
| High School | 31 (8.2%) | 30 (7.6%) | 36 (9.9%) |
| Some College | 148 (39.2%) | 160 (40.6%) | 137 (37.5%) |
| Bachelors | 148 (39.2%) | 145 (36.8%) | 147 (40.3%) |
| Masters | 39 (10.3%) | 53 (13.5%) | 32 (8.8%) |
| Doctorate | 12 (3.2%) | 5 (1.3%) | 13 (3.6%) |
| **Race/Ethnicity (%)** |  |  |  |
| White | 298 (78.8%) | 317 (80.5%) | 286 (78.4%) |
| Black | 32 (8.5%) | 19 (4.8%) | 22 (6.0%) |
| Asian | 22 (5.8%) | 21 (5.3%) | 18 (4.9%) |
| Mixed | 13 (3.4%) | 10 (2.5%) | 12 (3.3%) |
| Hispanic | 12 (3.2%) | 23 (5.8%) | 23 (6.3%) |
| Other | 1 (0.3%) | 4 (1.0%) | 4 (1.1%) |
| **Religion (%)** |  |  |  |
| None | 151 (39.9%) | 141 (35.8%) | 138 (37.8%) |
| Christianity | 124 (32.8) | 135 (34.3%) | 122 (33.4%) |
| Catholicism | 46 (12.2%) | 60 (15.2%) | 52 (14.2%) |
| Judaism | 8 (2.1%) | 8 (2.0%) | 10 (2.7%) |
| Islam | 7 (1.9%) | 4 (1.0%) | 3 (0.8%) |
| Hinduism | 6 (1.6%) | 2 (0.5%) | 1 (0.3%) |
| Prefer Not to Say | 15 (4.0%) | 18 (4.6%) | 8 (2.2%) |
| Other | 21 (5.6%) | 26 (6.6%) | 31 (8.5%) |
| **Income (%)** |  |  |  |
| Under $10,000 | 21 (5.6%) | 21 (5.3%) | 12 (3.3%) |
| $10,000 to 14,999 | 24 (6.3%) | 19 (4.8%) | 18 (4.9%) |
| $15,000 to 29,999 | 61 (16.1%) | 58 (14.7%) | 54 (14.8%) |
| $30,000 to 39,999 | 55 (14.6%) | 53 (13.5%) | 54 (14.8%) |
| $40,000 to 49,999 | 37 (9.8%) | 52 (13.2%) | 40 (11.0%) |
| $50,000 to 74,999 | 75 (19.8%) | 82 (20.8%) | 85 (23.3%) |
| $75,000 to 99,999 | 43 (11.4%) | 51 (12.9%) | 47 (12.9%) |
| $100,000 to 149,999 | 35 (9.3%) | 36 (9.1%) | 34 (9.3%) |
| $150,000 and over | 14 (3.7%) | 13 (3.3%) | 10 (2.7%) |
| Prefer Not to Say | 13 (3.4%) | 9 (2.3%) | 11 (3.0%) |
