# Supplementary material for: Can biased search results change people’s opinions about anything at all? a close replication of the Search Engine Manipulation Effect (SEME)
Source: PLoS One. 2024 Mar 26;19(3):e0300727. doi: 10.1371/journal.pone.0300727 (PMC10965084; doi:10.1371/journal.pone.0300727)
Supplement: S2 Table — (DOCX) [file pone.0300727.s003.docx]

**S2 Table: Demographics Analysis by Gender**

| **Experiment** |  | ***n*** | **MP (%)** | | **McNemar’s Test** | ***p*** |
| --- | --- | --- | --- | --- | --- | --- |
| **Artificial Intelligence** | **Male** | 165 | | 28.0 | 13.5 | < 0.001 |
|  | **Female** | 213 | | 23.0 | 9.76 | 0.002 |
|  | **Change (%)** | - | | 5.0 | - | - |
| **Fracking** | **Male** | 189 | | 23.9 | 6.25 | 0.012 |
|  | **Female** | 205 | | 38.5 | 19.12 | < 0.001 |
|  | **Change (%)** | - | | 14.6 | - | - |
| **Born Gay** | **Male** | 204 | | 14.7 | 3.77 | 0.049 |
|  | **Female** | 161 | | 21.7 | 7.04 | 0.007 |
|  | **Change (%)** | - | | 7.00 | - | - |
