# Supplementary material for: Can biased search results change people’s opinions about anything at all? a close replication of the Search Engine Manipulation Effect (SEME)
Source: PLoS One. 2024 Mar 26;19(3):e0300727. doi: 10.1371/journal.pone.0300727 (PMC10965084; doi:10.1371/journal.pone.0300727)
Supplement: S3 Table — (DOCX) [file pone.0300727.s004.docx]

**S3 Table: Demographics Analysis by Age**

| **Experiment** |  | ***n*** | **MP (%)** | | **McNemar’s Test** | ***p*** |
| --- | --- | --- | --- | --- | --- | --- |
| **Artificial Intelligence** | **≥ 30** | 205 | | 41.9 | 15.85 | < 0.001 |
|  | **< 30** | 173 | | 8.1 | 5.26 | 0.019 |
|  | **Change (%)** | - | | 33.8 | - | - |
| **Fracking** | **≥ 30** | 223 | | 30.3 | 16.48 | < 0.001 |
|  | **< 30** | 171 | | 31.7 | 8.31 | 0.004 |
|  | **Change (%)** | - | | 1.4 | - | - |
| **Born Gay** | **≥ 30** | 195 | | 18.7 | 7.35 | 0.011 |
|  | **< 30** | 170 | | 16.7 | 4.50 | 0.031 |
|  | **Change (%)** | - | | 2.0 | - | - |
