# Supplementary material for: Can biased search results change people’s opinions about anything at all? a close replication of the Search Engine Manipulation Effect (SEME)
Source: PLoS One. 2024 Mar 26;19(3):e0300727. doi: 10.1371/journal.pone.0300727 (PMC10965084; doi:10.1371/journal.pone.0300727)
Supplement: S4 Table — (DOCX) [file pone.0300727.s005.docx]

**S4 Table: Demographics Analysis by Education Level**

| **Experiment** |  | ***n*** | **MP (%)** | | **McNemar’s Test** | ***p*** |
| --- | --- | --- | --- | --- | --- | --- |
| **Artificial Intelligence** | **≥ Bachelor’s** | 199 | | 17.9 | 6.62 | 0.010 |
|  | **< Bachelor’s** | 179 | | 33.3 | 15.61 | < 0.001 |
|  | **Change (%)** | - | | 15.4 | - | - |
| **Fracking** | **≥ Bachelor’s** | 203 | | 30.2 | 15.61 | < 0.001 |
|  | **< Bachelor’s** | 191 | | 31.5 | 9.59 | 0.002 |
|  | **Change (%)** | - | | 1.3 | - | - |
| **Born Gay** | **≥ Bachelor’s** | 192 | | 21.4 | 11.64 | 0.001 |
|  | **< Bachelors** | 173 | | 13.9 | 1.89 | 0.167 (NS) |
|  | **Change (%)** | - | | 7.5 | - | - |
