# Supplementary material for: Can biased search results change people’s opinions about anything at all? a close replication of the Search Engine Manipulation Effect (SEME)
Source: PLoS One. 2024 Mar 26;19(3):e0300727. doi: 10.1371/journal.pone.0300727 (PMC10965084; doi:10.1371/journal.pone.0300727)
Supplement: S5 Table — (DOCX) [file pone.0300727.s006.docx]

**S5 Table: Demographics Analysis by Ethnicity**

| **Experiment** |  | ***n*** | **MP (%)** | | **McNemar’s Test** | ***p*** |
| --- | --- | --- | --- | --- | --- | --- |
| **Artificial Intelligence** | **White** | 298 | | 22.7 | 13.80 | < 0.001 |
|  | **Non-White** | 80 | | 33.3 | 7.56 | 0.004 |
|  | **Change (%)** | - | | 10.6 | - | - |
| **Fracking** | **White** | 317 | | 31.3 | 19.34 | < 0.001 |
|  | **Non-White** | 77 | | 29.2 | 4.9 | 0.021 |
|  | **Change (%)** | - | | 2.1 | - | - |
| **Born Gay** | **White** | 286 | | 17.9 | 13.79 | < 0.001 |
|  | **Non - White** | 79 | | 17.2 | 0.08 | 0.774 (NS) |
|  | **Change (%)** | - | | 0.7 | - | - |
