# Supplementary material for: Can biased search results change people’s opinions about anything at all? a close replication of the Search Engine Manipulation Effect (SEME)
Source: PLoS One. 2024 Mar 26;19(3):e0300727. doi: 10.1371/journal.pone.0300727 (PMC10965084; doi:10.1371/journal.pone.0300727)
Supplement: S1 Text — (DOCX) [file pone.0300727.s007.docx]

**S1 Text: Manipulation Power (MP) calculation**

100 * $\frac{p^{'}-p}{p}$

where *p* is the number of people who chose the opinion favored in the manipulation prior to that manipulation, and *p'* is the number of people who chose the opinion favored in the manipulation after that manipulation. Thus, MP can be defined as the post-manipulation percentage increase in the number of people choosing the opinion favored in the manipulation.
