# Supplementary material for: Can biased search results change people’s opinions about anything at all? a close replication of the Search Engine Manipulation Effect (SEME)
Source: PLoS One. 2024 Mar 26;19(3):e0300727. doi: 10.1371/journal.pone.0300727 (PMC10965084; doi:10.1371/journal.pone.0300727)
Supplement: S2 Text — (DOCX) [file pone.0300727.s008.docx]

**S2 Text: Artificial Intelligence Summary**

**Anti A.I.** Artificial intelligence, also known as A.I., refers to intelligent machines, computers, or software. A.I. can automate processes, and research is increasing its human-like capacities. As technology continues to improve, A.I. will grow more *dangerous* to humans. This view is generally considered to be **Anti A.I.**

**Pro A.I.** Artificial intelligence, also known as A.I., refers to intelligent machines, computers, or software. A.I. can automate processes, and research is increasing its human-like capacities. As technology continues to improve, A.I. will grow more *useful* to humans. This view is generally considered to be **Pro A.I.**
