# Supplementary material for: Can biased search results change people’s opinions about anything at all? a close replication of the Search Engine Manipulation Effect (SEME)
Source: PLoS One. 2024 Mar 26;19(3):e0300727. doi: 10.1371/journal.pone.0300727 (PMC10965084; doi:10.1371/journal.pone.0300727)
Supplement: S3 Text — (DOCX) [file pone.0300727.s009.docx]

**S3 Text: Fracking Summary**

**Pro-Fracking**. Fracking, also known as hydraulic fracturing, is a technique used to extract oil from rocks. It has potential economic benefits and is legal in most countries.
**Anti-Fracking**. Fracking, also known as hydraulic fracturing, is a technique used to extract oil from rocks. It has potential environmental risks and is regulated in some countries.
