# Supplementary material for: Can biased search results change people’s opinions about anything at all? a close replication of the Search Engine Manipulation Effect (SEME)
Source: PLoS One. 2024 Mar 26;19(3):e0300727. doi: 10.1371/journal.pone.0300727 (PMC10965084; doi:10.1371/journal.pone.0300727)
Supplement: S4 Text — (DOCX) [file pone.0300727.s010.docx]

**S4 Text: Sexual Orientation Summary**

**People choose to be gay**. Gay people, also referred to as lesbians or homosexuals, are people who only have romantic and sexual relationships with members of their own gender. Being gay is a *choice* people make, *not* a characteristic they are born with.
**People are born gay**. Gay people, also referred to as lesbians or homosexuals, are people who only have romantic and sexual relationships with members of their own gender. Being gay is *not a choice* people make, but a characteristic they are born with.
