# Supplementary material for: Can biased search results change people’s opinions about anything at all? a close replication of the Search Engine Manipulation Effect (SEME)
Source: PLoS One. 2024 Mar 26;19(3):e0300727. doi: 10.1371/journal.pone.0300727 (PMC10965084; doi:10.1371/journal.pone.0300727)
Supplement: S5 Text — (DOCX) [file pone.0300727.s011.docx]

**S5 Text: Request for Informed Consent**

By clicking continue I understand that I must be 18 or over to participate in this study, that my participation is voluntary, that I am free to withdraw at any time, that I am providing information anonymously and that demographic information collected is confidential and cannot be used to identify me. I agree to allow the data collected to be used for future research projects, and I understand that completion and submission of this survey implies my consent to participate in the present study.
